# Complementary feeding practice of mothers and associated factors in Hiwot Fana Specialized Hospital, Eastern Ethiopia

**DOI:** 10.11604/pamj.2014.18.143.3496

**Published:** 2014-06-17

**Authors:** Agumasie Semahegn, Gezahegn Tesfaye, Alemayehu Bogale

**Affiliations:** 1 College of Health and Medical Sciences, Haramaya University, Harar, Ethiopia

**Keywords:** Complementary feeding, initiation, associated factors, Ethiopia

## Abstract

**Introduction:**

Malnutrition remains one of the main public health problems. Over one third of under-five mortality is caused by under nutrition related to inadequate complementary feeding. This study was aimed to assess of complementary feeding practice and associated factors.

**Methods:**

Cross sectional study design was conducted to assess complementary feeding practice of mothers to their young children in Hiwot Fana specialized hospital in January 2013. Systematic sampling method was used to select 200 mothers to child pair to assess their timely initiation of complementary feeding and associated factors. Data were collected by using semi-structured questionnaire for face to face interview method. Descriptive statistics, binary and multiple logistic regressions were used for data analysis.

**Results:**

The prevalence of timely initiate of complementary feeding was 60.5%. Nineteen percent of mothers were initiate complementary before 6 months. The reason for too early initiation of complementary feeding was lack of knowledge and perceive inadequate breast milk production by mothers 17(47.2%), 11(30.6%) respectively. Mothers have male child three times more likely timely initiate complementary feeding than female child (AOR 2.9,95% CI 1.2-7.3). This might be due to traditional gender norm that discriminate female feeding “female eat little talk little” this might start at early age life.

**Conclusion:**

More than half of them initiated complementary feeding timely which was low. The main reason reported by the mothers for early initiation of complementary feeding was lack of knowledge. Mothers who have male child were three times more likely timely initiate complementary feeding than female child. Information about importance of timely initiation of complementary feeding should be implemented via information education and behavioral change communications, and integrating with health extension package is recommended.

## Introduction

There were approximately 10 million annual deaths of under-five year old children. Over one third of under-five mortality is caused by malnutrition related to inadequate complementary feeding. Initiate safe and nutritionally adequate complementary foods at 6 month is crucial to achieve optimal growth, development and health [1]. The world health organization has defined complementary feeding period as the period during which other foods or liquids are provided along with breast milk and any nutrient containing foods or liquids given to young children [2]. Optimal infant and young child feeding have single greatest potential impact on child survival. Complementary feeding starting at 6 months was third among 15 top ranked child survival interventions. Complementary feeding interventions alone were estimated to prevent almost one fifth of under five children mortality in developing countries [1, 3]. The most crucial time to meet child's nutritional requirements is the first 1,000 days. The child has increased nutritional needs to support rapid growth and development [4].

Improving quality of complementary food has been cited as one of the most cost effective strategies for improving health, reducing morbidity and mortality of young children. Nearly one third of child deaths could be prevented by optimal complementary feeding practices [1, 3, 5]. Approximately 50% of all childhood mortality were related to malnutrition, but also that the first 2 years of life represents a critical window of vulnerability [5]. The burden of under nutrition is still a major public health problem in the world. Almost 90% of the world's stunted children live in 36 developing countries. The potential negative impact of child under nutrition goes beyond the individual, affecting society and future generations [6]. Approximately more than 80% continued breast feeding into the child's second and third year in which it can significantly improves child survival and enhancing quality of life. More than 50% of infants aged 6-9 months had delayed introduction of complementary foods [7, 8].

A study done in china showed that 62.4% of mothers were introduced formula food, and 21.2% of them introduce cow milk from 6-12 months of age. However, 76% of mothers were introduced complementary food to their infant between 4 and 6 months of age. Timely complementary feeding was 41.6%. Maternal age, maternal education, employment and infant's sex were associated with early initiation of complementary feeding [9]. A study done in India found that nearly 48% of women were initiated complementary feeding during 6-9th month. More than one fourth of mothers give complementary food in 4-6th month. However, almost one in every ten mothers had initiated complementary food too late (12th month). Another study done in India found that more than three fourth of mothers had started complementary feeding at 6 month. However, 12% children had delayed complementary feeding. The most common reason given for the delayed introduction of complementary feed was mother felt their milk enough for baby [10, 11]. In developing world infant and child mortality remain quite high. In Pakistan, every eleventh child who is born alive dies before reaching one year of age [12].

A study done in Ghana revealed that 55% of the mothers had introduced other foods aside breast-milk within the ages of 3-4 months. However, 37.7% mothers introduced foods within the 5th and 6th months of their infants’ life. Mothers complementary feeding practice were influenced by family, friends and clinic based health workers in shaping current infant feeding practices. The age of mothers and their current feeding practices reveals an interesting trend. Meanwhile mothers with tertiary schooling were practicing formula feeding more than their counterparts. Mothers’ employment status was strongly associated with complementary feeding [13].

A study in Uganda indicated that almost half of women were initiate complementary feeding at six months. The most common foods offered to children before six months were water, cereal, traditional medicine and dry tea [14]. Three fourth of mothers were initiated complementary feeding around 4–6 months partial weaning [15]. In Ethiopia, complementary foods are not introduced in a timely fashion for many children, in which 14% of infants were on exclusive breast feeding up to 6-8 months of age [16]. Malnutrition remains one of the main public health problems in Ethiopia contributing to 53% of infant and child mortality. Approximately half of under five children were stunted in Ethiopia. The overall infant and young child feeding practices and child health remain weak [17, 18]. Complementary foods are not introduced in a timely fashion for all children. Only about half of children receive complementary foods at 6-9 months of age. Overall, only 4% of children ages 6-23 months are feed appropriately based on the recommended infant and young child feeding practices [18].

Appropriate child feeding has significant contribution on their growth and development. Evidence based feeding practice has crucial role on child mortality reduction. Ethiopia has been thriving to achieve the millennium development goal number 5. Therefore, determine the prevalence of complementary feeding practice and associating factors helps to show the progress of the country. The overall purpose of this study was to assess complementary feeding practice and associated factors among mothers in Hiwot Fana specialized hospital, Eastern Ethiopia.

## Methods

### Study setting and period

This study was conducted in Hiwot Fana specialized hospital, Harar town, Eastern Ethiopia in January, 2013. Harar town is located 526km from Addis Ababa to the Eastern part of Ethiopia. Harar town is the center of Harari people and national regional state. It has 19 urban kebele and 17 rural kebele (kebele is the smallest administrative structural unit in Ethiopia). According to the central statistics authority of Ethiopia 2007, Harari regional state has population of 183,415 of these 92,316 were male and 91,099 were female. There were 50,000 reproductive age group and 800 under five children. Hiwot Fana specialized hospital was established in 1941. It is referral hospital in Harar town and its surroundings which has been delivering health care services. Currently it is the teaching hospital of Haramaya University.

### Study Design and Sampling technique

Institution based cross sectional study design was conducted to assess complementary feeding practice of mothers. Sample size was determined by using single population proportion formula using assumption of 95% confidence level, 5% margin of error, prevalence timely initiation of complementary feeding practice was 14% [16]. For non responses, 10% were considered in the sample that yields 203 mothers-child pair. Systematic sampling method was used to select 203 mothers to child pairs according to their sequence visiting at the waiting room of the clinic. Mother-child pair who had revisit during the study period was skipped and used next client.

### Data collection instrument and data collection method

Data were collected by semi-structured questionnaire. The questionnaires were consisted of socio demographic characteristic of mothers, history of antenatal care visit and other variables that used to assess child feeding practice. Questionnaire was developed by adapting variables from different relevant literatures [13, 16, 18]. It was contextualized or modified to the study objectives and translated from English to Amharic language. Prior to data collection intensive training was given to data collectors about the questionnaire, interview technique and sampling procedure. Pretest was done at nearby hospital (Jegula hospital) and then necessary correction was taken accordingly. Data were collected using face to face interview method to assess complementary feeding practice of mothers.

### Data processing and analysis

Data were checked for incompleteness, inconsistency, edited, coded at field and at office. Then data were entered, cleaned and analyzed by using to SPSS window version 16.0. Descriptive statistics were used to compute prevalence of the timely initiation of complementary food and other variables. Binary logistic regression was used to determine association of the outcome variable with explanatory variables by 95% CI of crude odd ratio and p value. Variables had p-value less than 0.05 in the binary logistic regression analysis were entered to multiple logistic regressions. Multiple logistic regression statistical analysis (Hosmer-Lemeshow test of goodness of fit for the model) was used to identify independent associated factors of timely initiation of complementary feeding. Finally statistical association were declared at 95% confidence level, adjusted odd ratio and p value <0.05.

### Ethical clearance

Ethical clearance was obtained from college of health and medical sciences institutional ethics committee. Letter of cooperation was obtained from school of nursing and midwifery at Haramaya University. Permission was obtained from Hiwot Fana specialized hospital administrative office to collect data. Verbal consent was obtained from study participant, and confidentiality and the right of respondents not to participate were respected.

## Results

### Sociodemographic characteristics of mothers

Two hundred three mothers to child pairs were participate in this study that made 98.5% response rate. The mean age of mother's was 27.7 (+6.4) years old. Fifty one percent of mothers were in the age group of 25-34 years. Sixty seven percent of respondents were Muslim. Oromo 121(60.5%) was the largest ethnic group. Concerning the educational status of mothers, almost half 101(50.5%) of mothers had unable to read and write. Majority of mothers 177(88.5%) were married. Fifty two percent of mothers were urban resident (Table 1).

**Table 1:** Sociodemographic characteristics of mothers in Hiwot Fana specialized hospital, Harar, eastern Ethiopia, January 2013. (n=200)

Variable	Categories	Frequency	Percent
**Maternal age (in completed years)**	15-24	62	31.0
	25-34	102	51.0
	35-45	36	18.0
**Religion**	Muslim	134	67.0
	Orthodox	48	24.0
	Protestant	16	8
	Catholic	2	1.0
**Ethnicity**	Oromo	121	60.5
	Amhara	48	24.0
	Harari	20	10.0
	Others*	11	5.5
**Educational status of mothers**	Illiterate	108	54.0
	Grade 1-8	40	20.0
	Grade 9-12	34	17.0
	College or university	18	9.0
**Occupational status of the mother**	Farmer	42	21.0
	Government employee	22	11.0
	Daily laborer	17	8.5
	Merchant	24	12.0
	House wife	96	48.0
**Marital status of the mothers**	Married	177	88.5
	Divorced	17	8.5
	Others*	6	3%
**Residence**	Urban	104	52.0
	Rural	96	48.0
**Total**		200	100.0

* On marital status of mothers the others category include 2 single and 4 widowed mothers. On the other hand, the ethnicity variable on others category includes 4 Guraghe, 5 Tigre, 1 Somali and 1 Silte ethnic groups.

More than two third 139 (69.5%) of mothers had history of antenatal care during their pregnancy period of their youngest child. Among those mothers, 124(62.6%) had husband support during their antenatal care visits. Majority of them (59.0%) of women had more than 3 antenatal cares follow up during their pregnancy period. One hundred three (51.5%) of mothers had give their last birth at health institution. However, less than half 97(48.5%) had history of postnatal visit at six month (Table 2).

**Table 2:** Fathers characteristics with initiation complementary feeding to their young children attending Hiwot Fana specialized hospital, Harar, Ethiopia

Variables		Initiation of complementary feeding		P value
		Not timely (<6 and >6month)	Timely (at 6month)	
**Occupational status of father of a child**	Farmer	39(47.0%)	44(53.0%)	0.029
	Government worker	11(22.4%)	38(77.6%)	
	Daily laborer	12(50.0%)	12(50.0%)	
	Merchant	17 (38.6%)	27(61.4%)	
**Educational status of fathers**	Illiterate	42(50.0%)	42(50.0%)	0.001
	Grade 1-8	18(47.4%)	20(52.6%)	
	Grade 9-12	12(37.5%)	20(62.5%)	
	12+	7(15.2%)	39(84.8%)	
**ANC Visit**	Yes (139)	44(31.7%)	95(68.3%)	0.001
	No (61)	35(57.4%)	26(42.6%)	
**Minimum number of ANC visit (139)**	Once	4(26.7%)	11(73.3%)	0.077
	Twice	19(45.2%)	23(54.8%)	
	Three times+	21(25.6%)	61(74.4%)	
**Husband support during ANC (139)**	Yes	34(28.3%)	86(71.7%0	0.034
	No	10(52.6%)	9(47.4%)	
**Place of delivery**	Home	51(49.5%)	52(50.5%)	0.003
	Health institution	28(28.9%)	69(71.1%)	
**Post natal Visit**	Yes	29(29.9%)	68(70.1%)	0.007
	No	50(48.5%)	53(51.5%)	
**Sex of child**	Male	36(30.5%)	82(69.5%)	0.002
	Female	43(52.4%)	39(47.6%)	

p.value <0.05 consider as statistically significant

**Figure 1:**
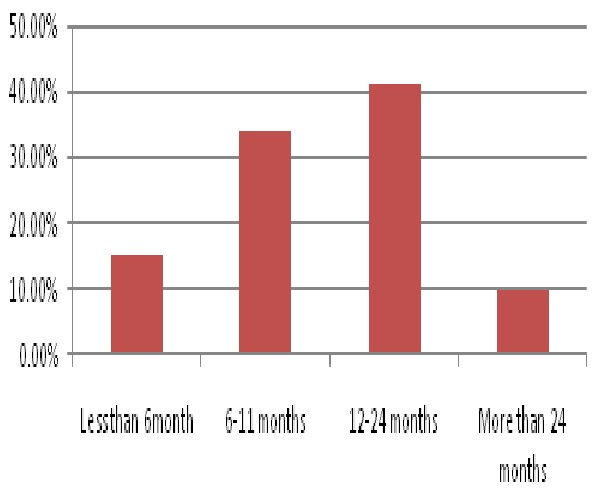
Breast feeding duration of young child among mothers attends at maternal and child clinic at Hiwot Fana specialized hospital, eastern Ethiopia, January 2013 (n=200)

The mean age of children was 25.4 (+14.9) months old. More than one third 78(39.0%) children were 25-59 months age. Others less than six months, 6-11months, 12-24 months were 8(4.0%), 53(26.5%) and 61(30.5%) respectively. Male were 118(59.0%) and 82(41.0%) were female. One hundred sixty four (82.0%) of mothers were give breast feeding to their children during the time of interview. Approximately two third 130(65.0%) of the mothers had give exclusive breast to their child for 6 months (Figure 1). One hundred seventy seven (88.5%) of children had history of vaccination. Of them 115(64.4%) had completed the whole dose of vaccine. Only 55(27.5%) of children had history of at least one episode of diarrhea for the last 12 months.

### Complementary Feeding Practice

**Figure 2:**
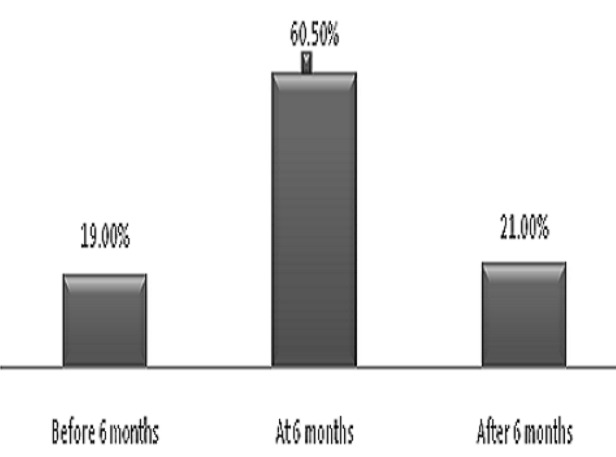
Mother's initiation of complementary feeding who had attend maternal and child clinic at Hiwot Fana specialized hospital, eastern Ethiopia, January 2013

Generally, 121(60.5%) of women were initiate complementary timely. Thirty eight (19.0%) of mothers were early initiate complementary feeding. The reason for to early initiation of complementary feeding (4-6months of age) was lack of knowledge 17(47.2%), inadequate breast milk production 11(30.6%) and due to far distance from health institutions 8(22.2%). Late initiation of complementary feeding was 79(39.5%). Both early initiation and late initiation were considered as not timely initiations which were 39.5% (Figure 2).

Fifty nine percent of women whose age from 15-24 years old were initiate timely, 64.7% of women whose age 25-34 years were initiate timely. There was consistent improvement of timely initiation of complementary with their educational status that illiterates, 1-8 grade completed, 9-12 grade completed and 12+ were 52.8%, 55.0%, 82.4%, and 77.8% respectively (p value < 0.007). However, 72.7% of government employee mothers were initiate complementary feeding to their young child. Regarding to occupation, only 66.7% of house wives were initiated complementary feeding timely. Urban resident women were initiate complementary feeding timely than rural residents 66.3% and 54.2% respectively. Approximately two third (64.4%) of married women were initiate complementary feeding timely. Child whose father government employee and have tertiary education were initiate complementary feeding timely (Table 3).

**Table 3:** Complementary feeding practice and sociodemographic characteristic of the mothers attending Hiwot Fana specialized hospital, Harar, Ethiopia, January 2013.

Variables		Complementary feeding initiation		P value
		Not timely (<6month & >6month)	Timely (at 6mn)	
**Age of mothers**	25-24	25(40.3%)	37(59.7%)	0.296
	25-34	36(35.3%)	66(64.7%)	
	35-45	18(50.0%)	18(50.0%)	
**Ethnicity**	Oromo	54(44.6%)	67(55.4%)	0.316
	Amhara	18 (33.3%)	32(66.7%)	
	Harari	6(30.0%)	14(70.0%)	
	Others *	3(27.3%)	8(72.7%)	
**Religion**	Muslim	58(43.3%)	76(56.7%)	0.072
	Orthodox	13 (27.1%)	35(72.9%)	
	Protestant	6 (37.5%)	10(62.5%)	
	Catholic	2	0	
**Educational status of mothers**	Illiterate	51(47.2%)	57(52.8%)	**0.007**
	Grade 1-8	18(45.0%)	22(55.0%)	
	Grade 9-12	6(17.6%)	28(82.4%)	
	12+	4(22.2%)	14(77.8%)	
**Occupational status of mothers**	Farmer	18 (43.9%)	23(56.1%)	0.065
	Government worker	6(27.3%)	16(72.7%)	
	Daily laborer	11(64.7%)	6(35.3%)	
	Merchant	12(50.0%)	12(50.0%)	
	Housewife	32(33.3%)	64(66.7%)	
**Residence**	Urban	35(33.7%)	69 (66.3%)	0.078
	Rural	44(45.8%)	52 (54.2%)	
**Marital status of mothers**	Married	63(35.6%)	114(64.4%)	0.007
	Divorced	12(70.6%)	5(29.4%)	
	Others	4(66.7%)	2(33.3%)	

Women who had history of antenatal care during their youngest child pregnancy period were initiate complementary feeding timely than their counter parts 68.3% and 42.6% respectively. Among women who give their both at health institution, 71.1% of them were initiate complementary feeding timely. Women who had post natal visit were more likely initiate complementary feeding timely.

Mothers were initiate complementary feeding liquid, semi solid, solid type food preparation 65(32.5%), 112(56.0%) and 23 (11.5%) respectively. Majority of women said, *“…usually prepare foods like porridge and soup using cow milk…. They did not accept cow milk as complementary food but they took as breast milk. That is why liquid food weaning is very small as compare to semi solid food weaning…”* However, 108(54.0%) of mothers were usually use milk and milk products to prepare complementary food. Thirty (18.5%) mothers used mashed vegetables. Fruits (banana, lemon), meat, egg and canned foods were 16(8.0%), 23(11.5%) and 15(7.5%) respectively. Fifty three percent of women were prepare special type of food for their child (6-59 months of age). About 152(76.0%) of the women were feed their young child 3-8 times per day. However, 35(17.5%) of women feed less than 3 times per day and 13(6.5% of mothers reported not make sure the frequency of feeding their child per day. Women reported their feeding practice during child hood illness period that 90(45.0%) of women feed their sick child accordingly, but 65(32.5%) of mothers feed as usual 3-8 times per day. Less than one fourth 45(22.5%) of mothers were feed their child less than 3times per day.

### Associated factors of complementary feeding practice

Variables like age, ethnicity, religion, marital status, educational status, occupational status, residence, antenatal care and husband support, educational status and occupational status of the father and sex of the child was enter to binary logistic regression to examine the association with timely initiation of complementary feeding. Illiterate women were less likely initiate complementary feeding than women who had 12+ educational level (p, 0.01), marital status of mother (p, 0.001), occupational status of the child's father (p, 0.04), as educational status of father increase the timely initiation of mothers improve (p, 0.002), mothers had history of antenatal care visit up to 3 times more likely initiate complementary feeding timely than their counter parts (OR 2.9, 95% CI 1.6-5.4). Mothers who had husband support were 3 times more likely initiate complementary feeding timely than their counter part (OR 2.8,95% CI 1.1-1.8). Mothers who had give birth at health institution were 2.4 times more likely initiate complementary food timely than mothers who had gave birth at home (OR 2.4,95% CI 1.3-4.3). Mothers who had history of post natal care visit were 2.2 times more likely initiate complementary feeding timely than mothers who had not history of postnatal visit (OR 2.2,95% CI 1.2-4.0). Mothers had male child were 2.5 times more likely initiate complementary feeding timely than mothers had female child (OR 2.5, 95%, CI:1.4-4.5) (Table 4). Finally the independent associated factors for timely initiation of complementary feeding of mothers were sex difference of the child. Mothers who had male child were 2.9 times more likely timely initiate complementary feeding than female child (AOR 2.9,95% CI 1.2-7.3). This might be due to traditional gender norm that discriminate female feeding “female eat little talk little” this might start at early age life (Table 5).

**Table 4:** Binary logistic regression of associated factors with complementary feeding practice of mothers attending Hiwot Fana specialized hospital, Harar, Ethiopia, 2013.

Variables		Complementary feeding initiation		COR 95% CI	P value
		Not timely (<6 & >6month)	Timely (at 6month)		
**Age of mothers**	25-24	25(40.3%)	37(59.7%)	1.5(0.6-3.4)	0.30
	25-34	36(35.3%)	66(64.7%)	1.8(0.8-3.9)	
	35-45	18(50.0%)	18(50.0%)	1.00	
**Ethnicity**	Oromo	54(44.6%)	67(55.4%)	1.00	0.32
	Amhara	18 (33.3%)	32(66.7%)	1.6(0.8-3.2)	
	Harari	6(30.0%)	14(70.0%)	1.9(0.7-5.2)	
	Others *	3(27.3%)	8(72.7%)	2.1(0.5-8.5)	
**Religion**	Muslim	58(43.3%)	76(56.7%)	1.00	0.28
	Orthodox	13 (27.1%)	35(72.9%)	2.1(1.0-4.2)	
	Protestant	6 (37.5%)	10(62.5%)	1.3(0.4, 3.7)	
	Catholic	2	0	0.00	
**Educational status of mothers**	Illiterate	51(47.2%)	57(52.8%)	0.3(0.1-1.0)	0.01
	Grade 1-8	18(45.0%)	22(55.0%)	0.3(0.8-1.4)	
	Grade 9-12	6(17.6%)	28(82.4%)	1.3(0.3-5.5)	
	12+	4(22.2%)	14(77.8%)	1.00	
**Occupational status of mothers**	Farmer	18 (43.9%)	23(56.1%)	0.6(0.3-1.4)	0.08
	Government worker	6(27.3%)	16(72.7%)	1.3(0.5-3.7)	
	Daily laborer	11(64.7%)	6(35.3%)	0.3(0.1-0.8)	
	Merchant	12(50.0%)	12(50.0%)	0.5(0.2-1.2)	
	Housewife	32(33.3%)	64(66.7%)	1.00	
**Residence**	Urban	35(33.7%)	69 (66.3%)	1.7(0.9-2.9)	0.08
	Rural	44(45.8%)	52 (54.2%)	1.00	
**Marital status of mothers**	Married	63(35.6%)	114(64.4%)	3.6(0.6-20.3)	0.01
	Divorced	12(70.6%)	5(29.4%)	0.8(0.1-6.1)	
	Others	4(66.7%)	2(33.3%)	1.00	
**Occupational status of father of a child**	Farmer	39(47.0%)	44(53.0%)	0.7(0.3-1.5)	0.04
	Government worker	11(22.4%)	38(77.6%)	2.2(0.9-5.4)	
	Daily laborer	12(50.0%)	12(50.0%)	0.6(0.2-1.7)	
	Merchant	17 (38.6%)	27(61.4%)	1.00	
**Educational status of fathers**	Illiterate	42(50.0%)	42(50.0%)	0.2(0.1-0.4)	0.002
	Grade 1-8	18(47.4%)	20(52.6%)	0.2(0.1-0.6)	
	Grade 9-12	12(37.5%)	20(62.5%)	0.3(0.1-0.9)	
	12+	7(15.2%)	39(84.8%)	1.00	
**ANC Visit**	Yes (139)	44(31.7%)	95(68.3%)	2.9(1.6-5.4)	0.001
	No (61)	35(57.4%)	26(42.6%)	1.00	
**Minimum number of ANC visit (139)**	Once	4(26.7%)	11(73.3%)	1.00	0.08
	Twice	19(45.2%)	23(54.8%)	0.4(0.1-1.6)	
	Three times+	21(25.6%)	61(74.4%)	1.1(0.3-3.7)	
**Husband support during ANC (139)**	Yes	34(28.3%)	86(71.7.0%	2.8(1.1-7.5)	0.04
	No	10(52.6%)	9(47.4%)	1.00	
**place of delivery**	Home	51(49.5%)	52(50.5%)	1.00	0.003
	Health institution	28(28.9%)	69(71.1%)	2.4(1.3-4.3)	
**Post natal Visit**	Yes	29(29.9%)	68(70.1%)	2.2(1.2-4.0)	0.007
	No	50(48.5%)	53(51.5%)	1.00	
**Sex of child**	Male	36(30.5%)	82(69.5%)	2.5(1.4-4.5)	0.002
	Female	43(52.4%)	39(47.6%)	1.00	

**Table 5:** Multiple logistic regression of associated factors with complementary feeding practice of mothers attending Hiwot Fana specialized hospital, Harar, Ethiopia, January 2013.

Variables		Complementary feeding initiation		COR 95% CI	AOR 95% CI	P value
		Not timely (<6 & >6mn)	Timely (at 6mn)			
Educational status of fathers	Illiterate	42(50.0%)	42(50.0%)	0.2(0.1-0.4)	0.3(0.04-2.2)	0.24
	Grade 1-8	18(47.4%)	20(52.6%)	0.2(0.1-0.6)	0.2(0.03-1.1)	
	Grade 9-12	12(37.5%)	20(62.5%)	0.3(0.1-0.9)	0.3(0.05-1.3)	
	12+	7(15.2%)	39(84.8%)	1.00	1.00	
ANC Visit	Yes (139)	44(31.7%)	95(68.3%)	2.9(1.6-5.4)	2.4(0.3-22.7)	0.45
	No (61)	35(57.4%)	26(42.6%)	1.00	1.00	
Husband support ANC (139)	Yes	34(28.3%)	86(71.7%	2.8(1.1-7.5)	1.1(0.3-3.9)	0.84
	No	10(52.6%)	9(47.4%)	1.00		
Place of delivery	Home	51(49.5%)	52(50.5%)	1.00	1.5(0.4-5.4)	0.54
	Health institution	28(28.9%)	69(71.1%)	2.4(1.3-4.3)	1.00	
Post natal Visit	Yes	29(29.9%)	68(70.1%)	2.2(1.2-4.0)	1.0(0.3-2.8)	0.97
	No	50(48.5%)	53(51.5%)	1.00	1.00	
Sex of child	Male	36(30.5%)	82(69.5%)	2.5(1.4-4.5)	2.9(1.2-7.3)	0.02
	Female	43(52.4%)	39(47.6%)	1.00	1.00	

## Discussion

This study determined prevalence of timely initiation of complementary feeding of women. Findings from this study showed that two third of the mothers had given exclusive breast to their child until six months and 35.0% of mothers initiated complementary feeding early (before 6 months) where one out of every five women started between 4-6 months. About 121(60.5%) of the women initiated complementary feeding timely. The main reason given by the mothers for early initiation of complementary feeding was lack of knowledge 17(47.2%). Most of the mothers, who were government employee, urban resident (P=0.078), had history of ANC follow up (P=0.45), more education (p value < 0.007), were married, give their birth at health institution (P=0.54), and had post natal visit (P=0.97) initiate complementary feeding timely than their others. Majority of the mothers 112(56.0%) initiate semi-solid complementary feeding liquid such as porridge and soup prepared from milk products. Sex difference of the child (AOR 2.9,95%CI 1.2-73) has significant association with timely initiation of complementary feeding on multivariate analysis.

In this study more than two third 139 (69.5%) of mothers had history of ante natal care visit during their pregnancy period of the youngest child from which the majority (59.0%) have had more than three times. This is lower than a study in Nepal in that 84.5% of the mothers have had at least one antenatal care visit and most of them had more than three times [19]. This may be due the low overall antenatal care coverage of in the country. One hundred three (51.5%) of mothers had given their last birth at health institution. One hundred sixty four (82.0%) of the mothers gave breast feeding to their children during the time of interview which is greater than the study in Nepal where 43.3% births occurred at health facilities [19]. This may be because of the difference in the studies setting and population. In this study 130(65.0%) of the mothers had given exclusive breast to their child up to six months which is quit higher than a study in Mauritius in which only 17.9% breastfed their children exclusively for the first 6months [15]. The 2011 Ethiopian DHS showed mothers exclusively breastfeed approximately half of children under six months (52.0%) which is relatively lower than the current study despite the HSDP IV targets an increase in the proportion of exclusively breastfed infants under age 6 months to 70 percent by 2015 [18].

The study also showed 55 (27.5%) of children had history of at least one episode of diarrhea for the last 12 months. Early introduction of liquids and solid foods at too early age increases the risk of diarrheal disease and important causes of infant and young children morbidity and mortality in Africa [20, 21]. Unlike this study where (35%) of mothers initiated complementary feeding early before 6 month, a study in Jimma [22] showed that 42.9% of the mothers introduced complementary food before 6 months. Relatively lower proportion of early initiation of complementary feeding may be explained by the fact that the presence of continuous effort towards improving children nutritional status in Ethiopia through nutritional education given by health extension workers. The main reason given for early initiation of complementary feeding (4-6months of age) was lack of knowledge 17(47.2%). A study in Jimma showed that 96(58.5%) gave good for growth as a main reason for early introduction of complementary feeding [22]. Timely introduction of solid foods remains an important factor for healthy infant growth. The study implies that premature introduction of complementary food was still of great concern in the present study.

The women who had history of antenatal care visit during their youngest child pregnancy period, who gave their birth at health institution and post natal visit initiate complementary feeding timely. A study in south India found out that timely initiation of complementary feeding is become higher with socio-economic status, birth order, place of delivery, maternal education [11]. Mothers prepare and initiate liquid, semi solid and solid type of complementary foods in 65(32.5%), 112(56.0%) and 23 (11.5%) respectively in which the majorities of them said they usually prepare foods like porridge and soup with cow milk but they did not accept cow milk as complementary food. However, 108(54.0%) of mothers are usually used milk and milk products to prepare complementary food. In line with this, findings from Jimma demonstrated that the majority 46.3 % of the mothers prepare the complementary food from cow milk [22]. This may be because cow milk is the most common animal product locally available for house hold consumption and believe that cow milk have high nutritional value. Another study in United Arab Emirate showed 30% of the infants were given non milk fluids. The majority of the infants (83.5%) received solid food before the age of 6 months [23].

In normal circumstance majority 152(76.0%) of the women feed their young child 3-8 times per day while 35(17.5%) of women feed less than 3 times per day. But a study in Nepal showed one third (33.27%) mothers fed their children with recommended frequency [24]. This indicates that there is improvement in the mother's awareness about frequent feeding of children in the study area and may be because of the inherent breast feeding culture in the society. In contrast to the other studies [15, 22, 24], Mothers who have male child were three times more likely to timely initiate complementary feeding than female child (AOR 2.9,95%CI 1.2-7.3). This might be due to traditional gender norm that discriminate female feeding “female eat little talk little” this might start at early age life.

### Strength and limitation of the study

The study used primary data that should consider as strength. But there can be recall bias among the participants and since it was facility based study, the findings may not be generalized. The study design was cross sectional so cause and effect relationship could not be established. So analytical study design is recommended to establish cause and effect relationship between associated factors and complementary feeding practice.

## Conclusion

This study showed that timely initiation of complementary feeding of mothers was low. More than half of them initiated complementary feeding timely. Less than a quarter of the women initiated complementary feeding early between 4-6 months. The main reason given by the mothers for early initiation of complementary feeding was lack of knowledge. Majority of the mothers initiate semi-solid complementary feeding liquid such as porridge and soup prepared from milk products. Mothers who have male child were three times more likely timely initiate complementary feeding than female child. Widespread information education communication and behavioral change communication activities on initiation of complementary feeding should be implemented in the maternal and child health unit of the hospital, and house to house by health extension workers and through integrating with health extension packages. Further community based research should be conducted.

## References

[1] UNICEF (2012). Infant and young child feeding, nutrition section program.

[2] Carlo A, Tamas D, Fewtrel M, Goulet O, Kolacek S, Koletzko B (2008). Complementary Feeding: Commentary by European Society for Pediatric Gastroenterology, Hepatology, and Nutrition. Journal of Pediatric Gastroenterology and Nutrition..

[3] International Baby Food Action Network (IBFAN) (2012). Report On the situation of infant and young child feeding in Liberia.

[4] United Nations Children's Fund (2013). Improving child nutrition: The achievable imperative for global progress.

[5] Nancy F Krebs, Hambidge K Michael (2007). Complementary feeding: clinically relevant factors affecting timing and composition. Am J Clin Nutr..

[6] Medhin G, Hanlon C, Dewey M, Alem A, Tesfaye F, Worku B (2010). Prevalence and predictors of undernutrition among infants aged six and twelve months in Butajira, Ethiopia. BMC Public Health..

[7] Lucy N Thairu, Gretel H Pelto, Nigel C Rollins, Ruth M Bland, Ncamisile N (2005). Sociocultural influences on infant feeding decisions among HIV-infected women in rural Kwa-Zulu Natal, South Africa. Maternal and Child Nutrition..

[8] Federal Ministry of Health, Family Health Department Ethiopia (2004). National Strategy for Infant and Young Child Feeding.

[9] Liubai L, Sujun L, Ali M, Ushijima H (2005). Feeding practice of infants and their correlates in urban areas of Beijing china. Pediatric international..

[10] Dakshayani B, Gangadhar MR (2008). Breast Feeding Practices among the Hakkipikkis: A Tribal population of Mysore district, Karnataka. Ethno-Med..

[11] Rao S, Swathi PM, Unnikrishnan B, Hegde A (2011). Study of complementary feeding practices among mothers of children aged six months to two years in coastal south India. Australasian Medical Journal AMJ..

[12] Syed Mubashir Ali (2001). Poverty and child mortality in Pakistan: Pakistan Institute of Development Economics Islamabad. Mimap Technical paper series NO 6. http://pide.org.pk/Mimap/Report06.pdf.

[13] Solomon Sika-Bright (2010). Socio-cultural factors influencing infant feeding practicies of mothers attending welfare clinic in Cape Coast. http://www.ifra-nigeria.org/IMG/pdf/Sika.pdf.

[14] Mokori A, Orikushaba P (2012). Nutritional status, complementary feeding practices and feasible strategies to promote nutrition in return children aged 6-23 months in northern Uganda. Afr J Clin Nutr..

[15] Ashmika M, Deerajen R, Pugo-Gunsam P, Rajesh J (2013). An Assessment of the Breastfeeding Practices and infant feeding pattern among mothers in Mauritius. Journal of Nutrition and Metabolism..

[16] Central Statistical Agency (Ethiopia) (2005). Ethiopia Demographic and Health Survey. http://www.prb.org/Publications/Articles/2006/EthiopiaDHS2005.aspx.

[17] USAID Investing in people: empowering new generations to improve nutrition and economic opportunities engine challenges in Ethiopia. http://www.idd.landolakes.com/stellent/groups/public/documents/web_content/ecmp2-0177333.pdf.

[18] Central Statistical Agency (Ethiopia) (2012). Ethiopia Demographic and Health Survey 2011.

[19] Khanal (2013). Determinants of complementary feeding practices among Nepalese children aged 6–23 months. BMC Pediatrics..

[20] WHO (2008). Strengthening action to improve feeding of infants and young children 6-23 months of age in nutrition and child health program.

[21] WHO (2009). Breastfeeding promotion and support in a baby-friendly hospital.

[22] Tamiru D, Aragu D, Belachew T (2013). Survey on the introduction of Complementary foods to infants within the first 6 Months and associated factors in rural communities of Jimma. International Journal of Nutrition and Food Sciences..

[23] Radwan (2013). Patterns and determinants of breastfeeding and complementary feeding practices of Mothers in the United Arab Emirates. BMC Public Health..

[24] Chapagain RH (2013). Factors affecting complementary feeding practices of Nepali Mothers for 6 to 24 months children. J Nepal Health Res Counc..

